# Activation of the mouse primary visual cortex by medial prefrontal subregion stimulation is not mediated by cholinergic basalo-cortical projections

**DOI:** 10.3389/fnsys.2015.00001

**Published:** 2015-02-09

**Authors:** Hoang Nam Nguyen, Frédéric Huppé-Gourgues, Elvire Vaucher

**Affiliations:** Laboratoire de Neurobiologie de la Cognition Visuelle, École D’optométrie, Université de MontréalMontréal, QC, Canada

**Keywords:** prefrontal cortex, cholinergic neurons, basal forebrain, visual cortex, neuronal activity, autometallography, thallium, immunocytochemistry

## Abstract

The medial prefrontal cortex (mPFC) exerts top-down control of primary visual cortex (V1) activity. As there is no direct neuronal projection from mPFC to V1, this functional connection may use an indirect route, i.e., via basalo-cortical cholinergic projections. The cholinergic projections to V1 originate from neurons in the horizontal limb of the diagonal band of Broca (HDB), which receive neuronal projections from the ventral part of the mPFC, composed of prelimbic (PrL) and infralimbic cortices (IL). Therefore, the objective of this study was to determine whether electrical stimulation of mice mPFC subregions activate (1) V1 neurons; and (2) HDB cholinergic neurons, suggesting that the HDB serves as a relay point in the mPFC-V1 interaction. Neuronal activation was quantified using c-Fos immunocytochemistry or thallium autometallography for each V1 layer using automated particle analysis tools and optical density measurement. Stimulation of IL and PrL induced significantly higher c-Fos expression or thallium labeling in layers II/III and V of V1 in the stimulated hemisphere only. A HDB cholinergic neuron-specific lesion by saporin administration reduced IL-induced c-Fos expression in layers II/III of V1 but not in layer V. However, there was no c-Fos expression or thallium labeling in the HDB neurons, suggesting that this area was not activated by IL stimulation. Stimulation of another mPFC subarea, the anterior cingulate cortex (AC), which is involved in attention and receives input from V1, activated neither V1 nor HDB. The present results indicate that IL and PrL, but not AC, stimulation activates V1 with the minor involvement of the HDB cholinergic projections. These results suggest a functional link between the ventral mPFC and V1, but this function is only marginally supported by HDB cholinergic neurons and may involve other brain regions.

## Introduction

The medial prefrontal cortex (mPFC) plays a ubiquitous role in decision making, attention and other cognitive processes in humans, primates and rodents. However, the structure-function relationship of the mPFC within its different subregions and with other brain structures remains to be better defined. For example, there is a functional influence of the prefrontal modulation of primary visual cortex (V1) activity (Kuo et al., 2014), but the anatomical relationship between the two cortical areas is not known.

The rat mPFC, as the homologs area in primates, the dorsolateral PFC, is involved in visual attention (Muir et al., 1996; Granon et al., 1998; Delatour and Gisquet-Verrier, 2000; Heidbreder and Groenewegen, 2003; Maddux and Holland, 2011) and cue guided behavior (Passetti et al., 2002; Kozak et al., 2006; Parikh et al., 2007). Both mechanisms may involve top-down control of the primary sensory areas, by modulating local responsiveness to afferent sensory stimuli. In this regard, mPFC has been shown to modulate V1 neuronal activity (Groenewegen and Uylings, 2000; Golmayo et al., 2003), but it does not directly project to V1 (Gabbott et al., 2005; Hoover and Vertes, 2007; Vogt and Paxinos, 2014). The mPFC in the rodent is composed of functionally and anatomically interacting subareas: the dorsal mPFC, which includes the medial agranular and anterior cingulate (AC) cortices, and the ventral mPFC (vmPFC), which includes the prelimbic (PrL) and infralimbic cortices (IL). The vmPFC is the major output region of the mPFC and shares anatomical connections with many parts of the brain, including the ventral tegmental area, the amygdala, several regions of the temporal lobe, the olfactory system, the hypothalamus, the hippocampal formation and the basal forebrain (BF), i.e., the diagonal band of Broca, substantia innominata and nucleus basalis magnocellularis (Vertes, 2004; Gabbott et al., 2005).

In V1, attentional mechanisms are partly mediated by acetylcholine (ACh), as shown in primates (Disney et al., 2007; Herrero et al., 2008) and rodents (Chudasama et al., 2004; Dalley et al., 2004). Recent studies linking the anatomical projection of the vmPFC to neurons of the BF (Zaborszky et al., 1997; Vertes, 2004) and the projection of those neurons to V1 (Gaykema et al., 1990; Laplante et al., 2005) proposed that the BF could be an anatomical and functional relay between the vmPFC and V1 (Golmayo et al., 2003). The horizontal limb of the diagonal band of Broca (HDB) is the main basalo-cortical cholinergic input to V1 (Mesulam et al., 1983; Gaykema et al., 1990; Laplante et al., 2005; Newman et al., 2012) and is involved in the neuromodulation of V1 activity (Dotigny et al., 2008; Kang and Vaucher, 2009; Kang et al., 2014). In addition to cholinergic cells, the BF contains GABAergic and glutamatergic cells with diverse projection patterns (Freund and Gulyás, 1991; Gritti et al., 1993, 2006; Zaborszky et al., 2012).

In the present study, the anatomo-functional interaction between the mPFC, HDB and V1 was examined in the mouse. Electrical stimulation of the two major mPFC subregions, IL/PrL and the AC, was performed, and the resulting neuronal activation was examined in V1 and the HDB by c-Fos immunocytochemistry and thallium (Tl) autometallography (TlAMG; Danscher, 1981; Goldschmidt et al., 2004; Stöber et al., 2014). The two complimentary techniques assess different aspects of neuronal activity. The contribution of cholinergic cells and fibers to the neuronal activation in V1 was confirmed by specific lesion of the cholinergic fibers in mice expressing green fluorescent EGFP protein under the control of the choline acetyltransferase (ChAT) promoter.

## Material and methods

### Animals

Adult mice expressing green fluorescent EGFP protein under the control of the ChAT promoter (B6g-tgRP23-EGFP, 30–40 g, founders from Jackson laboratory, Bar Harbor, ME, USA) and adult wild-type C57BL/6 mice (Charles rivers Canada, St-Constant, QC, Canada) were housed at a maximum of five per cage with food and water *ad libitum* in a temperature- and humidity- controlled room with a 12-h light/dark cycle. Experiments were conducted in accordance with the Canadian Council on Animal Care Guidelines and were approved by our local ethics committee for animal experimentation (protocol #12–172). EGFP colocalization with ChAT, a marker of cholinergic neurons, was ≥90%, as confirmed by ChAT immunostaining.

### Specific lesions of the cholinergic fibers

In the cholinergic deficit group, a specific lesion of the cholinergic fibers was performed by intraventricular injection of the neurotoxin p75-Saporin (Berger-Sweeney et al., 2001; Dotigny et al., 2008; Kang et al., 2014) 2 weeks prior to electrical stimulation of the mPFC. Mice were anesthetized with isoflurane (5% for induction and 2.5% for maintenance) with an oxygen flow of 1 l/min and placed into a stereotaxic frame (Kopf instruments). A hole in the skull was opened and a 33 gauge needle was inserted into the right lateral ventricle at: AP: −0.4; ML: 1.0 from Bregma; DV: −2.0 from dura matter (Berger-Sweeney et al., 2001). The neurotoxin p75-Saporin (mu p75-SAP, lot #94–69, Advanced Targeting Systems, San Diego, CA, USA) was administered in the right hemisphere (1 µl per site, 1 µg/µl in saline) at a flow rate of approximately 0.2 µl/min for 5 min. The skin was sutured and the animals were returned to their home cage for 14 days. Animals were then processed for electrical stimulation and further c-Fos immunostaining. The expression of EGFP (Figure 4A) or ChAT (Figure 5A) immunostaining was examined in the HDB neurons and their projections to V1 to verify the efficacy of the cholinergic lesion.

**Figure 1:**
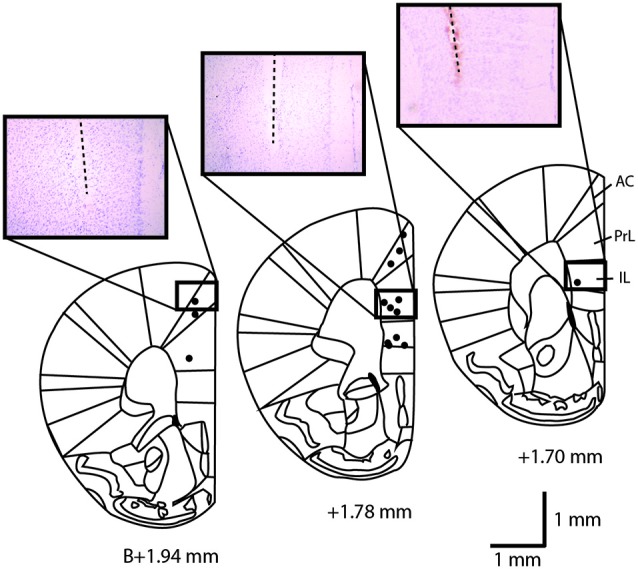
**Location of the stimulation sites in the mouse medial prefrontal cortex at +1.94, +1.78 and +1.70 mm anterior from Bregma**. Black dots represent the stimulated sites for the three mPFC stimulated subregions. Three examples of cresyl violet-stained sections at the level of the implantation sites are shown. The dotted lines indicate the location of the inserted stimulation electrode. AC, anterior cingulate cortex; IL, infralimbic cortex; PrL, prelimbic cortex. Scale bar = 1 mm.

**Figure 2:**
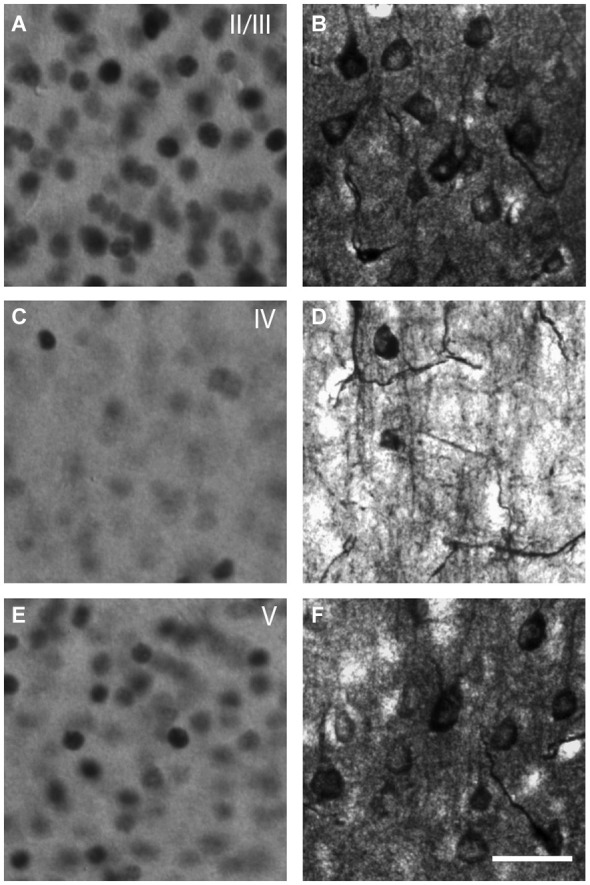
**Microphotographs representative of the neuronal activation in V1 detected by** **c-Fos immunocytochemistry (A,C,E) or thallium autometallography (B,D,F)**. c-Fos is a transcription factor expressed in the nucleus when neurons are activated. Thallium autometallography alternatively relies on the proportional uptake of K^+^ ions with neuronal activity. Note that c-Fos immunoreactivity is detected primarily in the layers II/III **(A)** and V **(E)** of V1 whereas TlAMG staining is observed in all layers **(B,D,F)** with layer IV **(D)** showing communicating fibers in TlAMG but not c-Fos **(C)**. Scale bar = 10 µm.

**Figure 3:**
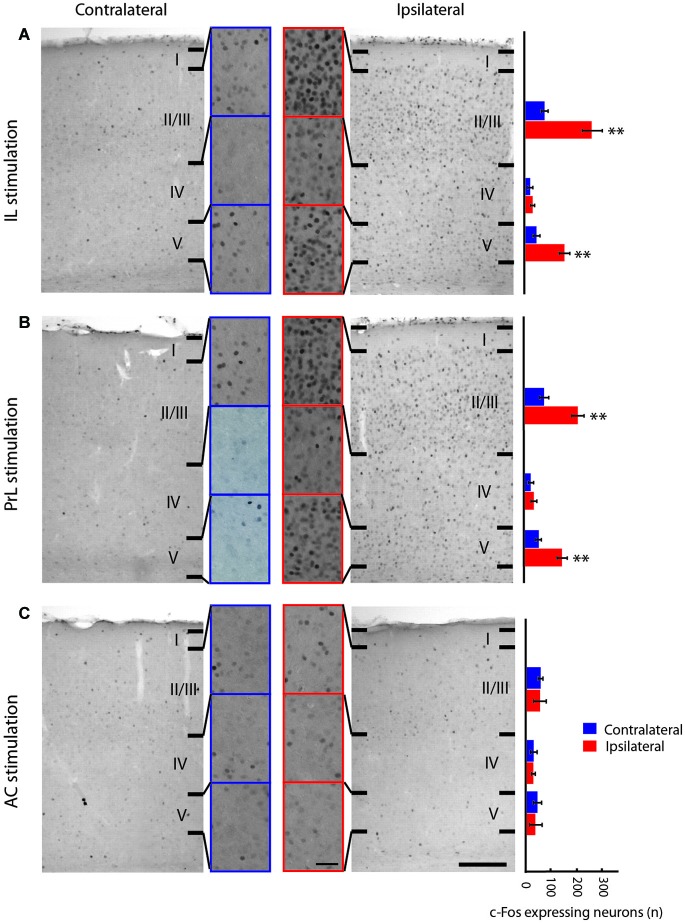
**Neuronal activity of the primary visual cortex induced by stimulation of medial prefrontal cortex subregions measured by c-Fos immunoreactivity. (A–C)**: Microphotographs of c-Fos immunostaining in the primary visual cortex following electrical stimulation of IL **(A)**, PrL **(B)** and AC **(C)** (right panels) compared to the non-stimulated hemisphere (left panels) at low and high (indents) magnification. Histograms of the number of c-Fos immunoreactive neurons (per 13 µm^2^ area) in the layers II/III, IV and V of the primary visual cortex after medial prefrontal cortex subregions stimulation (far right) are shown. The expression of c-Fos in V1 is increased in layers II/III and V following IL and PrL stimulation, but not following the AC stimulation. AC, anterior cingulate cortex; IL, infralimbic cortex; PrL, prelimbic cortex. Error bars = Standard Deviation (SD). Scale bar = 500 µm (panels) and 50 µm (indents). ** = *p* < 0.01.

**Figure 4:**
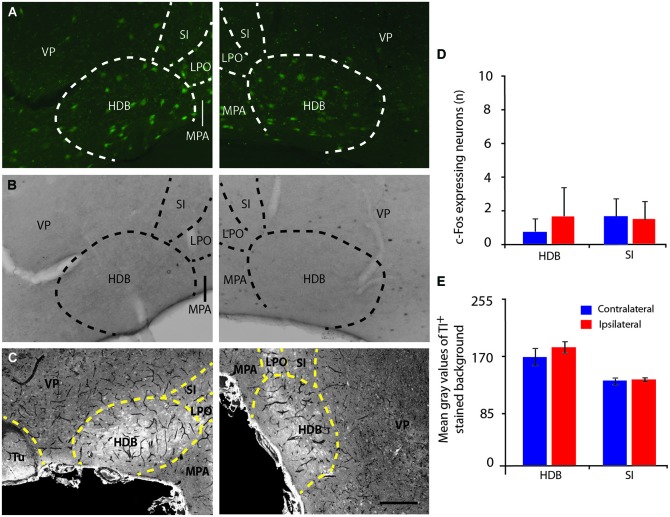
**Basal forebrain cholinergic neurons do not express c-Fos or thallium staining induced by IL stimulation. (A–C)** Microphotographs of ChAT:EGFP neurons (green) in the basal forebrain area with a high concentration of EGFP cells in the HDB **(A)**, c-Fos immunostaining **(B)** and thallium autometallography **(C)**. There was no staining of ChAT positive cells either by c-Fos (**B**, right panel) or thallium autometallography (**C**, right panel) in the basal forebrain of coronal brain sections at Bregma +0.62 mm following electrical stimulation of the IL (right panels) compared to the non-stimulated hemisphere (left panels). **(D,E)** Histograms of the number of c-Fos immunoreactive cells, note the near absence of c-Fos positive cells **(D)** or thallium stained cells **(E)** in the HDB and substantia innominata following stimulation of the IL, which suggest HDB and substantia innominata cholinergic neurons are not involved in V1 activation to vmPFC stimulation. HDB, Horizontal limb of the Diagonal band of Broca; LPO, Lateral Preoptic area; MPA, Medial Preoptic area; SI, Substantia Innominata; VP, Ventral Pallidum; Tu, Olfactory Tubercle. Delimitation of areas was designated in accordance with the atlas. Error bars = SD. Scale bar = 250 µm.

**Figure 5:**
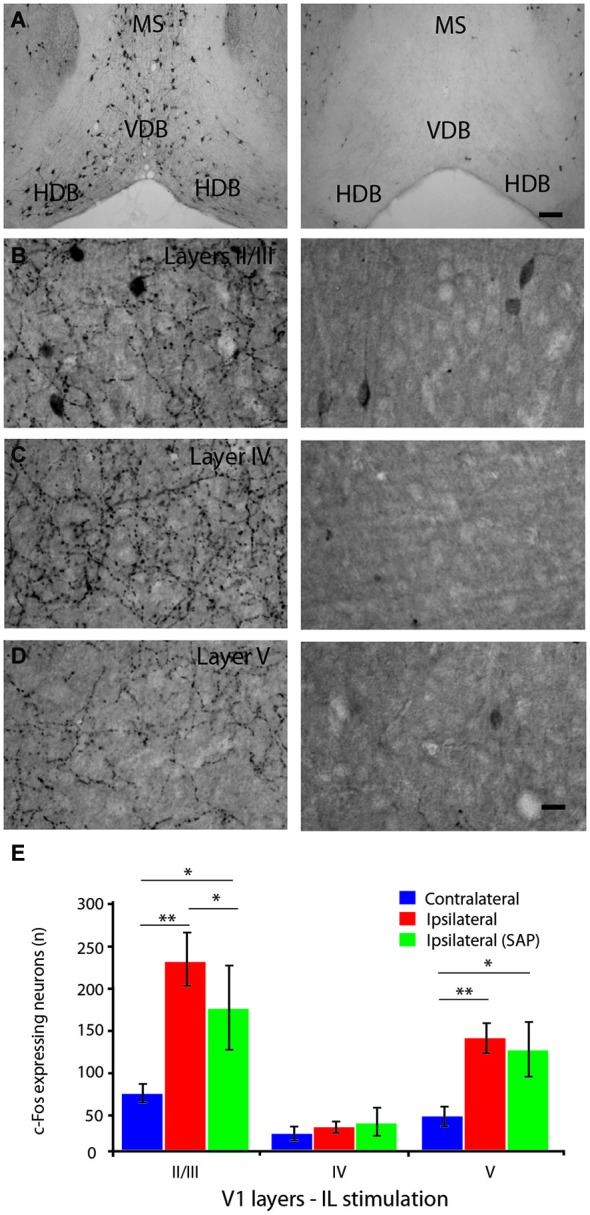
**Effect of the specific lesion of the cholinergic neurons on IL-induced neuronal activation of the primary visual cortex. (A)** ChAT-immunostained neurons in the HDB and surrounding areas in control (left panel) and saporin-lesioned mice (right panel). **(B,C)** ChAT-immunostained cholinergic fibers of the primary visual cortex in layer II/III **(B)**, layer IV **(C)** and layer V **(D)** for controls (left panels) and saporin-injected mice (right panels). Note the absence of cholinergic neurons in HDB, VDB and MS regions as well as cholinergic fibers in V1 after saporin injection. **(E)** Histograms of the number of c-Fos-immunoreactive neurons (per 13 µm^2^ area) in the layers II/III, IV and V of the primary visual cortex after IL stimulation in intact (red) or cholinergic lesioned (green) animals and in non-stimulated hemisphere (blue). The c-Fos expression in V1 was increased in the layers II/III and V following IL stimulation but the increase was attenuated in the layer II/III after lesion of cholinergic fibers. Scale bar **(A)** = 100 µm, **(D)** = 10 µm. Error bars = SD. * = *p* < 0.05, ** = *p* < 0.01.

### Electrical stimulation

Mice were anesthetized with urethane (1.2 g/kg, 10% in saline, i.p., Sigma-Aldrich, St. Louis, MO, USA). Electrodes were lowered at AP +1.78, L + 0.25 mm from Bregma, in IL, PrL or AC cortex (V −2.0, −1.25, −0.75 mm from dura matter, respectively). Five sham and five stimulated mice were used for each area of stimulation (IL, PrL, AC). In addition, five control mice and four mice with cholinergic lesions received unilateral electrical IL stimulation. These stimulation parameters were used for c-Fos and TlAMG (100 Hz for 0.3 s every 2 s, 50 µA) for either 15 (TlAMG) or 30 min (c-Fos). At the end of the experiment, an electrolytic lesion was performed and the electrode tip location was verified on cresyl violet stained coronal sections of the mouse brains. Stimulation site locations were reported on diagrams adapted from the brain atlas (Franklin and Paxinos, 2007; Figure 1).

### Neuronal activity measurement

Two complementary techniques were used to assess the neuronal activity of V1 and HDB neurons after mPFC stimulation: c-Fos immunoreactivity and TlAMG. c-Fos is a transcription factor involved in the transcription of synaptic genes. Recently activated cells overexpress the immediate-early gene *c-Fos*, the product of which can be visualized by immunocytochemistry. c-Fos has been used as a powerful metabolic marker of functional activity across brain areas, including in the visual system (Dragunow and Faull, 1989; Chaudhuri, 1997; Kaczmarek and Chaudhuri, 1997; Arckens et al., 2000; Zangenehpour and Chaudhuri, 2002). However, some cells, such as the GABAergic cells, rarely express c-Fos and the labeling is located only in the nucleus. TlAMG is based on the bioaccumulation of Tl^+^ ions that substitute potassium ions. Tl accumulates in cells and neurites during neuronal activation (Figure 2) through potassium channels. TlAMG Tl is then fixed by the perfusion of a sodium sulfide solution and is developed with silver for visualization under a microscope, as extensively described (Goldschmidt et al., 2004; Stöber et al., 2014). The use of TlAMG to visualize neuronal activation is similar to the 2-deoxyglucose method from Sokoloff (Goldschmidt et al., 2004), but with a subcellular resolution. The staining is not restricted to neurons but also stains neurites and blood vessels. In addition, the nature of the activated neurons can be assessed by their morphology. This technique has been used here to better visualize the possible activation of GABAergic BF neurons. A comparison of both stains is shown in Figure 2.

#### c-Fos Immunocytochemistry

Twenty-eight mice were used for c-Fos expression quantification. After electrical stimulation, urethane-anesthetized mice were restrained in darkness for 1 h and then transcardially perfused with 60 ml of 4% paraformaldehyde (PFA, Sigma-Aldrich). The brains were then post-fixed for 24 h in 4% PFA. Coronal brain sections (35 µm) covering the entire extent of the brain were sliced with a vibratome (Leica VT1000S), serially collected and stored in a phosphate buffer (PB)-glycerol based antifreeze solution for further use. Sections at 0.74 to 0.14 mm from Bregma were chosen for immunostaining in HDB and at −2.30 to −4.04 mm from Bregma for immunostaining in V1. Brain sections were incubated with rabbit anti-c-Fos (1:10000, Oncogene Research Products, San Diego, CA, USA) primary antibody or goat anti-ChAT (1:200, Millipore, Etobicoke, ON, Canada) in 0.12 M PBS, 0.25% triton X-100 (Sigma-Aldrich) and 0.5% donkey serum (Jackson Laboratories). Secondary biotinylated donkey anti-rabbit or anti-goat (Jackson Laboratories) antibodies were coupled with the ABC complex for detection. Slides were incubated for 1 h with the ABC complex and then visualized using the SG kit (Vector Laboratories).

Microphotographs of HDB and V1 were captured using a Leica DC500 camera. The counting of c-Fos-expressing cells was completed with ImageJ (*particle analysis*, pixel size 60) after thresholding on three slices at −2.54, −2.92 and −3.40 from Bregma for V1; at 0.74, 0.38 and 0.14 for HDB; at 1.34, 0.14 and −0.94 for the primary somatosensory cortex (S1); at −1.58, −1.82 and −1.94 for the posterior parietal cortex (PPC) and at 0.98, 0.26 and −0.22 for the midcingulate cortex (MC).

#### Thallium autometallography

Alternatively, the Tl-chelate solution was administered during mPFC stimulation to perform TlAMG. Ten mice were anesthetized with freshly prepared urethane (1.2 mg/kg) and positioned on the stereotaxic frame. Immediately before injection, equal volumes of 0.2% aqueous Tl (I) acetate (Sigma-Aldrich) solution and 0.2% sodium diethyldithiocarbamate trihydrate (Sigma-Aldrich) dissolved in 1.8% NaCl were mixed together in a syringe, forming the Tl diethyldithiocarbamate (TlDDC) complex. Mice were injected i.p. with 300 µL of 0.1% TlDDC in 0.9% saline immediately prior to the beginning of electrical stimulation of the IL.

After electrical stimulation, the animals were perfused (3 min) with a freshly prepared 0.05% sodium sulfide (Sigma-Aldrich) solution containing 100 mM PB at pH 7.4. The brains were then removed and post-fixed for 24 h in a 5% acrolein-PB solution at 4°C. The brains were then washed three times in 100 mM PB and placed in a 30% PB-sucrose solution for 48 h before freezing and storage at −80°C. Frozen brains were cut into 25 µm sections using a Microm cryostat (HM500OM), directly mounted on gelatin-coated glass slides and air-dried for 2 h.

After drying, slides were placed in 0.1 N HCl for 30 min to remove zinc sulfide from the sections, washed three times in distilled H_2_O, and bench air-dried again before staining. A developer solution was prepared for staining in accordance with standard autometallography methods as previously described by Goldschmidt et al. (2004, 2010). The sections were developed for 120 min in the dark followed with 10 min of washing under running tap water to stop development. The sections were then dried, dehydrated and cover-slipped with Permount (Fischer Scientific, Fair Lawn, NJ, USA).

Gray-scaled microphotographs were captured with a 40X Leica Fluotar objective quantification and a Leica DC500 camera. Neuron count and optical density quantification were performed using the mean gray values evaluated by ImageJ (Stöber et al., 2014). Three slices from each animal at −2.54, −2.92 and −3.40 mm from Bregma for V1 and at 0.74, 0.38 and 0.14 mm from Bregma for HDB and substantia innominata were selected for neuron count and/or mean gray values analysis in layers II/III, IV and V of V1. Five equivalent sample areas from each animal/slice/layer were compiled for analysis. High gray scale values correspond to low staining intensity in TlAMG, i.e., low neuronal activity.

### Statistical analysis

Mann-Whitney U tests were computed to compare neuronal activity in the contralateral and ipsilateral hemispheres of the same animals and between groups for each layer using SPSS 19 (SPSS Inc., Chicago, IL, USA) for c-Fos expression, TlAMG neuron count and TlAMG mean gray values. Statistical analyses were calculated for activity in the HDB and V1.

## Results

### Effect of the mPFC stimulation on the expression of c-Fos

Electrical stimulation of the IL subarea of the vmPFC (Figure 3) evoked increased c-Fos expression in the ipsilateral V1 in layers II/III (*p* = 0.009) and layer V (*p* = 0.009), but not in layer IV (Figure 3A) nor in any of the BF regions, including the HDB and substantia innominata (Figures 4B,D). These structures were also activated by the stimulation of the PrL cortex, which also induced a similar neuronal activation pattern in V1 as IL stimulation (Figure 3B). Thus, both structures were considered analogs, as suggested previously (Vogt and Paxinos, 2014), and further analysis of vmPFC stimulation effects was performed with IL stimulation only. IL-induced c-Fos activation was also increased in other regions of the cortex ipsilateral to the stimulation, including the MC (*p* = 0.009), the S1 (*p* = 0.009), the PPC (*p* = 0.009), the primary and secondary auditory cortices, the temporal association cortex, the ectorhinal cortex, the piriform cortex, the medial and lateral septum nuclei, the bed nucleus of the stria terminalis, the preoptic areas and the paraventricular and medial thalamic nuclei (Figures 7A–C). Electrical stimulation of the AC subarea of the dmPFC did not evoke any change in c-Fos expression in any layers of V1 (Figure 3C) or in the HDB or other BF structures.

**Figure 6:**
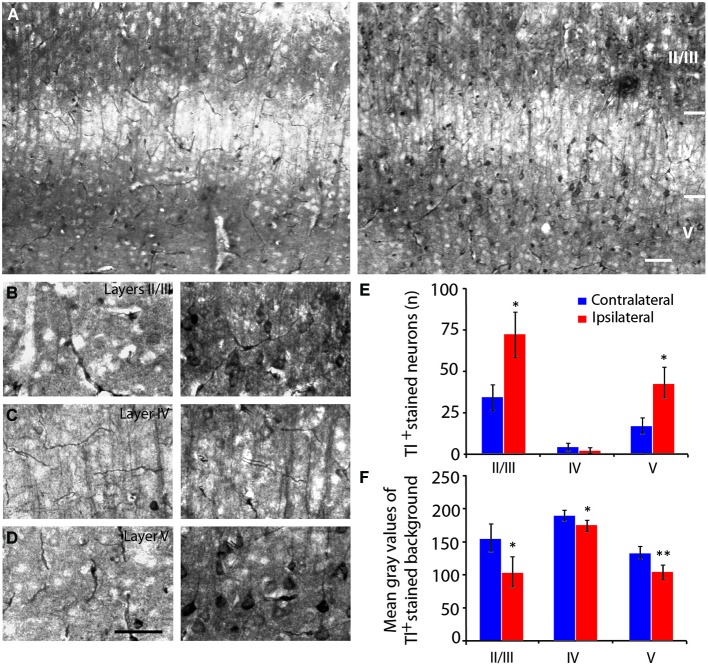
**Neuronal activity of the primary visual cortex measured by thallium autometallography. (A)** Thallium staining in the primary visual cortex after infralimbic cortex stimulation (right panel) compared to non-stimulated hemisphere (left panel). **(B–D)** Details of thallium staining in V1 layers II/III **(B)**, IV **(C)** and V **(D)** of non-stimulated (left panels) and stimulated hemispheres (right panels). Because Tl^+^ accumulation is correlated with increased activity, the darker neurons (and the background neurites) indicate stronger activity. Quantification of thallium-stained neurons **(E)** and mean optical density values **(F)** in primary visual cortex at −3.28 mm from Bregma following infralimbic cortex stimulation. High gray scale values correspond to low staining intensity. In the primary visual cortex, neuron bodies were strongly stained in layers II/III (**B**, right panel) and V (**D**, right panel) as well as pass-through fibers in layer IV (**C**, right panel) after stimulation of the infralimbic cortex. Scale bars = 10 µm. Error bars = SD. * = *p* < 0.05, ** = *p* < 0.01.

**Figure 7:**
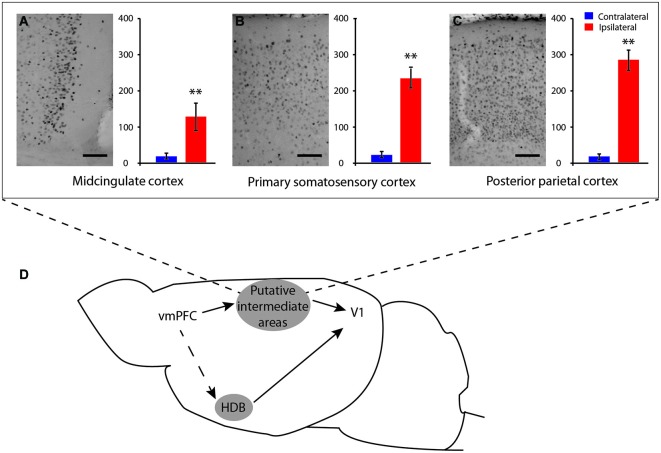
**Schematic representations of possible pathways from vmPFC to V1**. Microphotographs of putative IL-induced c-Fos-immunostaining in intermediate cortical areas with corresponding quantification of c-Fos neurons number **(A)** midcingulate cortex (B-0.26 mm); **(B)** primary somatosensory cortex (B-0.96 mm) and **(C)** posterior parietal cortex (B-1.82 mm). Possible communication pathways between these cortices and the vmPFC, HDB, and V1 are shown in **(D)**. These cortice might be possible relays in the communication between vmPFC and V1. S1, sensory motor cortex; MC, mid cingulate cortex; PPC, posterior parietal cortex. Scale bar = 250 µm. Error bars = SD. ** = *p* < 0.01.

### Effect of cholinergic fiber lesions on mPFC stimulation-induced neuronal activity

To further evaluate the role of HDB cholinergic neurons in the IL-evoked increase of V1 neuronal activity, a specific lesion of the cholinergic neurons was performed with p75-saporin intraventricular injection. The lesion showed a complete elimination of ChAT-EGFP- or ChAT-immunolabeled cell bodies in the BF region, including HDB (Figure 5A). The lesion was specific to BF neurons because ChAT-EGFP neurons were observed in other brain regions, such as the striatum and pedonculopontine nucleus, which is consistent with the literature (Berger-Sweeney et al., 2001; Dotigny et al., 2008). The ChAT fibers innervating V1, which predominantly originate from the HDB, were also lesioned (Figures 5B,C,D).

c-Fos expression induced by IL electrical stimulation was significantly reduced in layers II/III (*p* = 0.025) but not layer V in the lesioned group compared to non-lesioned group (Figure 5E), suggesting a contribution of the cholinergic innervation of V1 in the mPFC-induced neuronal activity in V1.

### Effect of the mPFC stimulation on the thallium autometallography staining

IL stimulation evoked an increase in the number of Tl-positive neurons in the layers II/III (*p* = 0.021) and V (*p* = 0.021) of V1 (Figures 6A–E). In addition, analysis of the mean optical density or gray level histogram distribution representative of the neuronal activity in both the cell body and the neurites showed increased potassium intake in the layers II/III (*p* = 0.016), IV (*p* = 0.024) and V (*p* = 0.009) following IL stimulation (Figure 6F). There were very few stained neurons in the HDB or in either hemisphere (Figures 4C,E), suggesting a non-activation of the HDB and substantia innominata neurons in accordance with the c-Fos immunostaining.

## Discussion

The present study demonstrates that activation of the IL and PrL mPFC subregions, but not the AC, induced neuronal activation in layers II/III and V of V1. This neuronal activity was partly dependent on the integrity of the cholinergic innervation of V1, although the activity of HDB neurons was not readily apparent, as evaluated by c-Fos or Tl staining. This suggests a marginal contribution of the cholinergic system in mPFC-induced V1 activation either locally or through the PFC-HDB-V1 neuroanatomical pathway. The distribution of the effect of IL stimulation includes other cortical or subcortical regions, including the somatosensory cortex and amygdala, which could also be involved in the indirect activation of the V1 by the IL.

### Organization of the mPFC

This study demonstrates a regional organization of the mouse mPFC with distinct effects on V1, i.e., IL/PrL, but not AC stimulation, activates V1 neurons. As IL and PrL stimulation induced similar neuronal activation patterns, it also suggests that IL and PrL are equivalent in terms of activating V1 and could be grouped as a single functional unit, i.e., vmPFC, as previously proposed (Passetti et al., 2002; Vertes, 2004). The distinction of vmPFC from dmPFC, which have different functional roles and communication networks (Muir et al., 1996; Passetti et al., 2002; Guillem et al., 2011; Cassaday et al., 2014; Feja and Koch, 2014), is thus supported by our results. Particularly the sensory output function of the vmPFC compared to the input function of the dmPFC may be underlined by the influence of IL/PrL on V1 shown here. This duality agrees with studies of vmPFC lesions, which affect choice accuracy, latency and perseverative response (Muir et al., 1996; Feja and Koch, 2014) as well as attentional flexibility and inhibition (Murphy et al., 2005; Gisquet-Verrier and Delatour, 2006) that may be related to the control of neuronal firing in V1 by IL/PrL. In contrast, lesions of the rat anterior dmPFC induce anticipatory responses in the five-choice serial reaction time task (Muir et al., 1996), suggesting a role related to behavior inhibition rather than visual processing.

It has been suggested that mPFC function during behavioral tasks involving attention, vision and motivation may be dependent on interaction between mPFC subareas (Heidbreder and Groenewegen, 2003; Cassaday et al., 2014; Vogt and Paxinos, 2014). Our results do not confirm a possible interaction between IL/PrL and AC, although the AC is interconnected with PrL and IL in rats (Vogt and Miller, 1983; Bedwell et al., 2014; Vogt and Paxinos, 2014). There was no contralateral activation after IL stimulation, although the IL projects to the contralateral mPFC (Vertes, 2004). These results may be due to experimental conditions, i.e., passive and localized stimulation.

### Distribution of the vmPFC-induced activity in V1

Layers II/III and V of the V1 ipsilateral to the stimulated side were activated by unilateral IL/PrL electrical stimulation, as assessed by immunocytochemistry and autometallography. The activation of layer II/III neurons may indicate a cortico-cortical component because feedback cortical projections predominantly reach cortical layer II/III (Wang and Burkhalter, 2007; Gao et al., 2010; Andermann et al., 2011; Wang et al., 2011). The activation of layer IV measured by densitometry (decreased TlAMG mean optical density) only reflects the increased staining of pass-through fibers between layers II/III and V because no neuron cell bodies were stained with TlAMG in layer IV. The pattern of activation in V1 thus does not appear to result from a thalamo-cortical pathway. Layer V activation indicates a cortico-thalamic output component induced by the stimulation. Thus, according to the pattern of V1 neuronal activation, which is similar to an attentional top-down control (Gilbert and Li, 2013), the vmPFC appears to exert feedback influence on layer II/III neuronal processing in V1, which is further transmitted to layer V. Given that the layer II/III neurons integrate visual information and transfer the visual information to higher visual areas, it is reasonable to suggest that our results correspond to an attention-related modulatory role of V1 processing by the IL.

### Possible neural pathways between vmPFC and V1

The ipsilateral cerebral distribution of IL stimulation effects as well as activation of S1, MC, and PPC areas suggests that IL-V1 interaction could be mediated by cortico-cortical pathways (Figure 7D), although activation of fibers of passage could not be discarded. IL is known to send subcortical projections to the amygdala, the diagonal band, the preoptic areas, the hypothalamus and to some areas of the thalamus (Vertes, 2004), which will also be discussed.

#### Involvement of cortico-cortical connections

Our results first suggest that the IL may exert its V1 activation function through the MC. IL stimulation induced strong ipsilateral activation of area 29b—MC cortex. The stimulation of this structure induces neuronal activation of V1 (Zhang et al., 2014). Our results are supported by a recent study showing that the IL/PrL area, which corresponds to area 24a, projects to the MC area 29b, which itself projects to V1 (Vogt and Paxinos, 2014). However, it should be mentioned that according to the same study (Vogt and Paxinos, 2014), the AC also sends projections to area 29b, but in our study, the AC did not induce V1 activation, although a bilateral weaker activation of 29b was observed. This absence of V1 activation may be related to the strength of the PFC interconnections as discussed above.

The present results also show strong activation of S1 and PPC following both PrL and IL electrical stimulation, which could also be relay areas in the activation of V1. It has been previously shown that mPFC stimulation—although more dorsal than our stimulation location—induces an evoked potential (Golmayo et al., 2003) or release of ACh linked to attentional mechanisms (Nelson et al., 2005), in the PPC. Connections between the mPFC and sensory-motor cortex (primary motor and somatosensory cortices) have also been documented in rats (Bedwell et al., 2014) as well as long-range cortico-cortical projections from the somatosensory cortices to the visual cortex in the mice (Charbonneau et al., 2012; Wang et al., 2012, 2014). In primates, it has been shown that the interaction between prefrontal areas and V1 is mediated through parietal cortico-cortical top-down pathway connections (Gilbert and Li, 2013). The implication of the parietal cortex in attention is also well documented relative to the role of saccadic control during attention by fronto-parietal activation of the PFC (Rizzolatti et al., 1987). Thus, it is possible that an analogs system of feedback from the mPFC to V1 via the parietal cortex occurs in rodents (Figure 7).

#### Involvement of the subcortical structures, including the basal forebrain

It is possible that the amygdala plays a role in the interaction of the vmPFC with the HDB and V1. The amygdala activation observed in the present study is supported by strong projections from the mPFC to the amygdala (Vertes, 2004). Projections from the amygdala to V1 (Senn et al., 2014) and the HDB (Luiten et al., 1987) have also been described. It is known that the pulvinar is involved in top-down control of V1 in primates (Purushothaman et al., 2012); however, the equivalent nucleus in rodents—i.e., lateral posterior thalamic nucleus—is not activated by any of the stimulation sites in the present study—although these neuron may express c-Fos in other activation conditions (Sonoda et al., 2013).

Because lesioning cholinergic neurons in the HDB partially reduced layer II/III V1 activation, it might suggest that these cholinergic neurons could participate in the modulatory effect of IL on V1, but are not strongly involved. ACh can modulate intracortical connectivity and functional organization (Groleau et al., 2014) and participate in numerous cognitive and visual functions, including attention and perceptual learning (Kang et al., 2014). IL is the prefrontal subregion with the strongest reciprocal connections with the HDB. Thus, HDB neurons, which project to V1, could be a relay point between IL and V1. This schematic has been proposed previously by Golmayo et al. (2003) and furthered by others (Nelson et al., 2005). However, our present experiments did not detect any cells activated in the HDB, neither cholinergic nor non-cholinergic. Additionally, there was no activation in BF regions adjacent to the HDB (e.g., substantia innominata, vertical diagonal band of Broca or nucleus basalis magnocellularis). This result is surprising given that spiking activity has been detected in the BF after IL/PrL stimulation (Gyengési et al., 2008) and the pattern of c-Fos activation of V1 is similar to the pattern observed after electrical stimulation of the BF (Kocharyan et al., 2005; Kang et al., 2014). This absence of staining is not due to technical limitation because c-Fos staining has already been shown in BF cells (McKenna et al., 2009) and TlAMG is also negative for any potassium fluxes in the BF during IL stimulation. It is possible that the stimulation paradigm of the IL used here may inhibit cholinergic neuron output to the V1 through inhibitory input from BF GABAergic interneurons, which receive projections from the mPFC (Zaborszky et al., 1997). However, the low activation of the entire HDB compared to surrounding areas, as seen by the TlAMG optical density measurement, do not support an involvement of GABAergic neurons. Alternatively to the involvement of BF neurons, the cholinergic lesion could have affected layer II/III microfunction by depleting ACh in V1. In agreement, we previously proposed that local neuronal activity in V1 could trigger local release of ACh from surrounding cholinergic axons (Laplante et al., 2005). Moreover, PFC-elicited V1 activity could involve local release of ACh (Lambe et al., 2003; Parikh and Sarter, 2008) independently from BF activation. In agreement with a modulatory role of the mPFC on the ACh efflux in V1, the mPFC lesion prevented the ACh release elicited by visual stimulation in V1 (Rasmusson et al., 2007)—although the pathways of this inhibition were not clear. Thus, the stimulation of mPFC in our study may have involved cholinergic transmission within V1.

In conclusion, the top-down control of V1 by mPFC appears to be exerted by the IL and PrL subregions rather than by the AC. This IL/PrL functional connection to V1 is only marginally supported by HDB cholinergic basalo-cortical projections and may require other brain regions, including other cortical areas, such as the mid- and posterior-cingulate, somatosensory cortex or PPC. This functional connection may be related to visual attention processes.

## Conflict of interest statement

The authors declare that the research was conducted in the absence of any commercial or financial relationships that could be construed as a potential conflict of interest.
